# Intracellular trafficking and cellular uptake mechanism of PHBV nanoparticles for targeted delivery in epithelial cell lines

**DOI:** 10.1186/s12951-016-0241-6

**Published:** 2017-01-03

**Authors:** Juan P. Peñaloza, Valeria Márquez-Miranda, Mauricio Cabaña-Brunod, Rodrigo Reyes-Ramírez, Felipe M. Llancalahuen, Cristian Vilos, Fernanda Maldonado-Biermann, Luis A. Velásquez, Juan A. Fuentes, Fernando D. González-Nilo, Maité Rodríguez-Díaz, Carolina Otero

**Affiliations:** 1 Center for Integrative Medicine and Innovative Science, Facultad de Medicina, Universidad Andrés Bello, Santiago, Chile; 2 Escuela de Bioquímica, Facultad de Ciencias Biológicas, Universidad Andrés Bello, Santiago, Chile; 3 Center for Bioinformatics and Integrative Biology, Facultad de Ciencias Biológicas, Universidad Andrés Bello, Echaurren #183, 8370071 Santiago, Chile; 4 Departamento de Ciencias Físicas, Facultad de Ciencias Exactas, Universidad Andrés Bello, Santiago, Chile; 5 Laboratorio de Genética y Patógenesis Bacteriana, Facultad de Ciencias Biológicas, Universidad Andrés Bello, Santiago, Chile; 6 Escuela de Química y Farmacia, Facultad de Medicina, Universidad Andrés Bello, Santiago, Chile

## Abstract

**Background:**

Nanotechnology is a science that involves imaging, measurement, modeling and a manipulation of matter at the nanometric scale. One application of this technology is drug delivery systems based on nanoparticles obtained from natural or synthetic sources. An example of these systems is synthetized from poly(3-hydroxybutyrate-co-3-hydroxyvalerate), which is a biodegradable, biocompatible and a low production cost polymer. The aim of this work was to investigate the uptake mechanism of PHBV nanoparticles in two different epithelial cell lines (HeLa and SKOV-3).

**Results:**

As a first step, we characterized size, shape and surface charge of nanoparticles using dynamic light scattering and transmission electron microscopy. Intracellular incorporation was evaluated through flow cytometry and fluorescence microscopy using intracellular markers. We concluded that cellular uptake mechanism is carried out in a time, concentration and energy dependent way. Our results showed that nanoparticle uptake displays a cell-specific pattern, since we have observed different colocalization in two different cell lines. In HeLa (Cervical cancer cells) this process may occur via classical endocytosis pathway and some internalization via caveolin-dependent was also observed, whereas in SKOV-3 (Ovarian cancer cells) these patterns were not observed. Rearrangement of actin filaments showed differential nanoparticle internalization patterns for HeLa and SKOV-3. Additionally, final fate of nanoparticles was also determined, showing that in both cell lines, nanoparticles ended up in lysosomes but at different times, where they are finally degraded, thereby releasing their contents.

**Conclusions:**

Our results, provide novel insight about PHBV nanoparticles internalization suggesting that for develop a proper drug delivery system is critical understand the uptake mechanism.

**Electronic supplementary material:**

The online version of this article (doi:10.1186/s12951-016-0241-6) contains supplementary material, which is available to authorized users.

## Background

Nanotechnology is the science of engineering materials and systems on a molecular scale. Its application to medicine, “nanomedicine”, has enabled the development of nano-sized drug-delivery vehicles. These nanocarriers are generally <200 nm in size and have the ability to carry and deliver therapeutics to discrete sites into the cells [1].

Due to their small size, increased stability and sustained drug release properties, biodegradable polymeric nanocarriers display several advantages, being more effective for cancer treatment than other nanoparticles (e.g. metallic ones) [2]. Nanoparticles are being developed for in vivo tumor imaging, targeted drug delivery and biomolecular profiling of cancer biomarkers. Biodegradable polymeric nanoparticles (NPs) have been shown promissory as controlled drug delivery systems, showing high therapeutic potential [3]. Currently, delivery technologies using cell-targeting [4, 5] or specific targeting of organelles inside a cell [6] are becoming increasingly important as an area of scientific investigation. In cancer, polymeric NPs can be used to deliver chemotherapeutics towards tumor cells, with high efficiency and reduced cytotoxicity on healthy tissues [6–8]. The activity of the drug carried by the NPs depends on how and where the NPs are disassembled. The mechanism of NPs endocytosis and trafficking is still a subject of controversy [9–11].

Among polymeric NPs, (3-hydroxybutyric acid-co-3 hydroxyvaleric acid) (PHBV) constitutes a promissory alternative, more accessible and cheaper than similar polymers such as poly (lactic-co-glycolic acid) (PLGA); the last one has been widely used in drug delivery in different cancer types and have an FDA approval [12, 13]. PHBV is a biodegradable and biocompatible polyester, composed of two monomers: hydroxybutyrate and hydroxyvalerate and, since it is produced by bacteria or eukaryotic cells, it can be synthesized in large scales [14]. However, at present, little is known about its uptake and distribution into mammalian cells [14].

In the case of hydroxybutyrate-composed homopolymers (PHB), it has been described that is primarily found complexed with other molecules, and can be found in several subcellular organelles of eukaryotic organisms [15] or associated with proteins [16]. A previous study has suggested that PHB is associated in granules that accumulate in the cytoplasm, similar to the behavior in bacteria, which might constitute an ‘energy reservoir’. In the same study, the colocalization of PHV granules with organelles was inspected in U87 cells, showing no remarkable colocalization with lysosomes, mitochondria, or endoplasmic reticulum [17].

PHBV copolymer reduces the chain packing and decreases crystalline melting point, due to the ethyl chain of the valerate, which increases flexibility, impact strength, and ductility in comparison to hydroxybutyrate homopolymer [18]. Furthermore, previous studies have revealed the usefulness of PHBV nanoparticles in the encapsulation of paclitaxel drugs, protecting this anticancer agent against premature degradation, by allowing 48 h of toxicity protection. Other applications of PHBV in medicine include a formulation loaded with antibiotics for veterinary uses, which allows protecting the drug for inactivation [19].

Previous work demonstrates that most NPs, including those formed from biodegradable polymers such as PLGA, are taken up by an endocytic process, and their uptake is concentration- and time-dependent [12, 20, 21]. PLGA NPs for delivery of therapeutics are of particular interest due to their biocompatibility, biodegradability and ability to maintain therapeutic drug levels for sustained periods of time [22]. However, further investigation is required to determine the mechanisms and therapeutic potential of intracellular targeting of nano-delivery systems in vivo for the goal of an anticancer vaccine [14].

Other studies on PLGA uses for targeting have shown that the intracellular fate of nanoparticles may be altered by their surface decoration with a targeting molecule. In the case of PLGA, previous studies in smooth muscle cells have revealed that these NPs can undergo exocytosis in about 65%; meaning that the NPs are effectively internalized, but they escape from the cells before releasing their content [23]. The fraction of NPs that escapes from the endosomes to the cytosolic compartments was shown to be retained inside cells, delivering the encapsulated agent slowly [24]. The polymer matrix prevents the degradation of the drug and the duration and levels of drug released from the NPs can be easily modulated by altering the formulation. Meanwhile, in hepatoma cells, it has been described that PLGA NPs are taken up by the classical clathrin pathway and that they effectively escape from lysosomes and contribute to enhancing the efficiency of intracellular delivery and tumor therapy [25].

In summary, discovering the intracellular fate of NPs and the investigation of their endocytic mechanism is particularly significant with the aim of using them as drug carriers, since in some cases, they may be taken back to the extracellular media (via recycling endosomes), or they may be degraded in lysosomes or be trapped in an organelle, without releasing its content at the desired site [12, 26, 27]. Furthermore, escaping from the endosomes to reach other organelles is another important issue to be addressed.

The present manuscript aims to provide insights about the possible routes of internalization and escaping from the endosomal sorting pathway of PHBV nanoparticles, in two different cell lines: in HeLa (cervix cancer) and SKOV-3 (ovarian cancer). HeLa cells was chosen because its endocytic machinery is very well characterized, and SKOV-3 cells because these cells are challenging due to their common chemoresistance [28] in order to investigate how a possible drug treatment (encapsulated in a nanoparticle) may stay inside the cell, where normally drugs are pumped out.

Several compartments, such as early endosomes (Transferrin Conjugate or EEA1 antibody), late endosomes (Lysotracker or Rab7 antibody) and lysosomes (Lysotracker or LAMP1 antibody), were chosen for this investigation, as they are involved in transport, destination, release, and degradation of soluble and membrane-bound macromolecules [29]. Endosome and lysosome formation require microtubules to form a network in the cytoplasm, whereas actin generates forces to induce membrane invaginations [30, 31], thereby microtubules and actin cytoskeleton were also analyzed.

These methods may contribute to the development of PHBV NPs as drug carriers, enabling to enhance the delivery of chemotherapeutics inside the cells.

## Methods

### Materials

Poly (3-hydroxybutyric acid-co-hydroxyvaleric acid) (PHBV) with 12 wt% PHV and polyvinyl alcohol (PVA) (average mol wt. 30,000–70,000) and glutathione were obtained from Sigma-Aldrich (St. Louis, MO, USA). Nile Red 552/636 was purchased from Invitrogen (Carlsbad, CA, USA). Dichloromethane (DCM), dimethyl sulfoxide (DMSO) and methanol were purchased from Merck (Darmstadt, Germany). The primary antibody Anti-EEA1 (Early Endosome Antigen 1) was purchased in Santa Cruz Biotechnology (Santa Cruz, CA, USA), the anti-Caveolin-1 and anti-LAMP1 (Lysosomal-associated membrane protein 1) were obtained from Abcam (Cambridge, USA). The secondary antibodies Alexa Fluor 488 Donkey Anti-Mouse IgG (H + L), and Alexa Fluor 488 Goat Anti-Rabbit IgG (H + L) were purchased in Molecular Probes by Life Technologies (Carlsbad, CA, USA). Additionally, membrane glycoproteins marker Wheat Germ Agglutinin, Alexa Fluor 555 Conjugate (WGA), actin (F-actin) Alexa Fluor 488 phalloidin, LysoTracker® Red DND-99 and Hoechst 33342 were obtained from Molecular Probes by Life Technologies (Carlsbad, CA, USA). Fluoromount G was purchased in Electron Microscopy Sciences (Hatfield, PA, USA), and EZ-Link® NHS-SS-Biotin was obtained from Pierce Biotechnology (Rockford, IL, USA).

### Preparation of PHBV nanoparticles

PHBV nanoparticles (NPs) were formulated via a modification of the double emulsion (w1/o1/w2) solvent-evaporation method [19]. Briefly, 400 µL of distilled water were added to 1 mL of a solution of 3 mg/mL of PHBV (Sigma-Aldrich, Co., St. Louis, MO, USA) in dichloromethane (Merck KGaA, Darmstadt, Germany). The first emulsion (w1/o1) was prepared by sonication in an ultrasonic processor equipped with a microtip probe for 40 s at 100% in an ice bath. The water-in-oil emulsion was further emulsified by sonication under the same conditions in 4 mL of an aqueous solution of 5 mg/mL PVA (w2). This w1/o1/w2 emulsion was immediately poured into a beaker containing 20 mL of a 0.5 mg/mL PVA solution. The mixture was stirred with an overhead propeller for 12 h under a flow hood, and the solvent was allowed to evaporate. The remaining organic solvent and free molecules were removed by passing the particle solution 3 times through an Amicon Ultra-4 centrifugal filter. Finally, NPs were concentrated by centrifugation and resuspended in 500 μL of phosphate buffered saline (PBS, pH 7.4) for further use. A modification of the technique described was used for the synthesis of functionalized nanoparticles with a marker. The same procedure was followed, except the aqueous phase (W1) was replaced by 100 μL of a solution of fluorescein isothiocyanate (FITC) 1 mg/mL; 100 μL of a solution of Nile Red (RN) 1 mg/mL; or 5 μL of the LysoTracker® Red DND-99 fluorescent probe.

### PHBV nanoparticles characterization by dynamic light scattering (DLS)

Each preparation was suspended in 1 mL of PBS pH 7.4 and nanoparticles diameter (nm), polydispersity coefficient (PdI) and zeta potential (mV) were determined by light scattering technique using a Zetasizer Nano-ZS (Malvern Instruments Ltd., UK).

### Transmission electron microscopy (TEM)

NPs structure was also characterized using transmission electron microscopy. One drop of the NP sample was placed onto an ultra-thin Lacey carbon-coated 400-mesh copper grid and allowed to dry at room temperature for 10 min prior to image acquisition, ensuring no more than 1 min of electron beam exposure to the sample. TEM images were acquired using an LVEM5 electron microscope (Delong Instrument, Montreal, Quebec, Canada) at a nominal operating voltage of 5 kV. The small volume of the vacuum chamber in the LVEM5 microscope facilitates rapid sample visualization within 3 min before observation. The low voltage used delivers high contrast in soft materials (up to 20-fold) compared with high-voltage electron microscopes, which use accelerating voltages of approximately 100 kV; this procedure facilitates the emission of staining procedures and allows the direct visualization of biological samples. Digital images were captured using a Retiga 4000R camera (QImaging, Inc., USA) at its maximal resolution.

### Cell culture

The Human cervical adenocarcinoma HeLa and the human ovarian adenocarcinoma SKOV-3 were obtained from the American Type Culture Collection (ATCC® CCL-2™, and ATCC® HTB-77™ respectively). The cells were maintained in Dulbecco’s High Glucose Modified Eagle’s Medium (DMEM) supplemented with 10% v/v fetal bovine serum, 1 mM sodium pyruvate and 1% v/v penicillin–streptomycin (Hyclone™ Laboratories, Inc., South Logan, UT, USA), and incubated at 37 °C and 5% CO_2_. Peripheral blood mononuclear cell (PBMC) were obtained using a Ficoll® density gradient, and cultured in Roswell Park Memorial Institute Medium (RPMI) 1640, which was supplemented with 10% v/v fetal bovine serum and 1% penicillin–streptomycin (Hyclone™ Laboratories, Inc., South Logan, UT, USA) and incubated at 37 °C and 5% CO_2_.

### Flow cytometry

HeLa and SKOV-3 cells (2 × 10^5^ cells/well) were seeded separately in 6-well plates and incubated for 48 h. Subsequently, to evaluate time-dependent intracellular incorporation, a solution of PHBV-RN 100 μg/mL was added in culture medium for 5, 15, 30 min, 1 and 2 h. To assess the dependence of the concentration, different concentrations (1, 10, 100, 500 and 1000 μg/mL) of a PHBV-RN solution were added and incubated for 2 h. Additionally, to evaluate the dependence of the energy, cells were incubated at 4 °C or with a sodium azide (AS) solution 1 mg/mL (Sigma-Aldrich, Co., St. Louis, MO, USA) for 1 h respectively. Then, a PHBV-RN solution (100 μg/mL) was added and incubated for 2 additional hours. After the incubation time, cells were washed 3 times with cold PBS, detached from the culture plate with PBS-EDTA 0.2% (w/v), incubated for 15 min at 37 °C, then washed twice with buffer FACS (PBS-2% FBS) and resuspended in 500 μL of PBS. Finally, the fluorescence intensity of cells containing fluorescent nanoparticles was analyzed using a flow cytometer BD Accuri C6™ and v1.0 software (BD™, Franklin Lakes, NJ, USA).

### Immunofluorescence

Cell lines were plated at a confluence of 70–80% in 24-well plates with coverslips. A PHBV-FITC or PHBV-RN nanoparticles solution was added and incubated for different times. Then, cells were washed 2 times with PBS, fixed with paraformaldehyde (PFA) 4% for 10 min, washed 3 times with PBS and then permeabilized with Triton X-100 0.1% by 10 min. Then, cells were washed 2 times with PBS and then cells were incubated with a primary antibody (1:100) for 1 h at RT. Then, cells were washed 5 times again with PBS and incubated with a secondary antibody (1:500) for 1 h at RT. Nucleic acid marker Hoechst 33342 (1:1000) was added for 15 min and finally cells were washed 5 times with PBS. Coverslips were mounted on a slide using Fluoromount G mounting medium for subsequent microscopic observation. Slides were observed using a BX61 fluorescence microscope (Olympus Corp., Tokyo, Japan), coupled to an ORCA-R2 (Hamamatsu Photonics KK, Japan) camera. Images were analyzed by Dimension cellSens v1.7.1 software (Olympus Corp., Tokyo, Japan). Colocalization percentages were based on the merge between the two channels red and green. For this, we use Image J v1.48 software using the plugin Colocalization Threshold which creates a threshold for pixels of the red and green channels. Pixels below this threshold are ignored for the quantification of colocalization.

### Reducible biotin assay

To analyze the involvement of proteins involved in endocytosis of nanoparticles, we performed an experiment using reducible biotin. When biotinilated proteins are internalized, they become resistant to extracellular reducing solution, allowing distinction between endocytosed and not endocytosed proteins. Experiment were performed at 4 °C. Cells were grown to 70–80% confluence in 6-well plates and washed 3 times with cold PBS. Biotin (0.5 mg/mL) were incubated for 20 min two times with gentle shaking. Free biotin was blocked adding a solution of 50 mM NH_4_Cl for 10 min, and then cells were washed 3 times with PBS. For the reduction, a solution containing glutathione 50 mM, 90 mM NaCl, 10% FBS, 1 mM MgCl_2_, 0.1 mM CaCl_2_ and 60 mM NaOH to a final pH of 7.2–7.4 was used. Cells were incubated with the solution two times for 30 min at 4 °C with gentle shaking. Then cells were incubated with lysis buffer (containing antiproteases) with gentle agitation for 30 min at 4 °C. The obtained extract was centrifuged for 30 min at 14,000 rpm at 4 °C and the pellet was discarded. Proteins were quantified using Qubit® 2.0 Fluorometer. 30 µg of protein were denatured in protein loading buffer and incubated at 95 °C for 5 min. Samples were loaded onto a polyacrylamide gel 12% acrylamide-bis.

### Western blot

Electrophoresis was performed at 120 V constant current in running buffer in a vertical electrophoresis chamber model Mini PAGE System. After electrophoresis, proteins were transferred to a preactivated PVDF membrane in 100% methanol. The transfer was carried out in a transfer model system Trans-Blot® Turbo™. The PVDF membrane was blocked with gentle agitation for 2 h at room temperature in PBS-Tween 20 0.5, 10% glycerol, glucose 1 M BSA 3% skim milk and 1%. After blocking, the membrane was washed 4 times for 15 min with PBS-0.05% Tween 20 and subsequently incubated for 2 h at room temperature with streptavidin horseradish peroxidase conjugated (1:1000) in PBS with 0.5% Tween 20, 10% glycerol, 1 M glucose, 0.3% BSA. Finally, the membrane is washed 4 times for 15 min with PBS-0.05% Tween 20. For the development of the membrane substrate SuperSignal West Femto Chemiluminescent substrate (Thermo Fisher Scientific Inc., Rockford, IL, USA) was used according to manufacturer’s instructions. Development was carried out in the PHOTO/Analyst® Luminary/FX® Systems equipment and using the FOTO/Analyst® PC Image v5.0 (Fotodyne Inc., Hartland, WI, USA) software.

### MTT assay

Toxicity of the nanoparticles was determined using the 3-(4,5-dimethylthiazol-2-yl)-2,5-diphenyltetrazolium bromide (MTT) cell viability assay. MTT is a yellow compound that when reduced by functioning mitochondria, produces purple formazan crystals that can be measured spectrophotometrically. For this purpose, HeLa cells were incubated with different concentrations of PHBV solution for 24 h at 37 °C and 5% CO_2_. After incubation time, MTT (Sigma-Aldrich) was dissolved in phosphate buffered saline (PBS) to a concentration of 5 mg/mL and further diluted in culture medium (1:11). Cells were incubated with this MTT-solution for 3 h under normal culture conditions. Afterwards 155 μL of the solution were rejected and 90 μL of DMSO were added. To completely dissolve the formazan salts plates were incubated for 10 min on a shaker and afterwards quantified by measuring the absorbance at 535 nm with a ELISA microplate reader. Cell viability was calculated as percentage of surviving cells compared to untreated control cells.

## Results and discussion

### Characterization of PHBV nanoparticles

Double emulsion solvent evaporation method was used to formulate four preparations of PHBV nanoparticles: PHBV-empty (control), FITC-loaded PHBV NPs (PHBV-FITC), Nile Red-loaded PHBV NPs (PHBV-RN), and Lysotracker-loaded PHBV NPs (PHBV-Lysotracker®). This w/o/w method was selected due of the broad experience of our group in the formulation of NPs, and the incorporation of dyes in the aqueous or organic phase was according to its solubility. The characterization of size and morphology of NPs was performed using dynamic light scattering (DLS) and by transmission electron microscopy (TEM). Table 1 describes the diameter (nm), polydispersity index (PdI), and the Zeta Potential (mV) obtained of NPs, and Additional file 1: Figure S1 exhibits a graphical representation of the data analyzed statistically. The size presented in all formulations of NPs was homogenous with a diameter of ~200 nm, stable in time (Additional file 2: Figure S2), and similar to the size of paclitaxel-loaded PHBV nanoparticles synthesized previously by our group [32].

**Table 1:** Nanoparticle physicochemical characterization: size, zeta potential and polydispersity index (PdI) (n = 5)

	Size (nm)	PdI	Zeta potential (mV)
PHBV	200.2 ± 3.66	0.133 ± 0.020	−11.7 ± 1.97
PHBV-FITC	201.7 ± 10.4	0.100 ± 0.023	−9.60 ± 1.25
PHBV-RN	206.0 ± 3.37	0.204 ± 0.043	−7.96 ± 0.41
PHBV-Lysotracker®	201.5 ± 7.19	0.206 ± 0.021	−5.16 ± 1.99

Studies have revealed a direct relationship between the size of the NPs and the endocytic pathway. Particles with a size less than 200 nm were internalized into non-phagocytic, murine melanome cells B16-F10 via clathrin-mediated endocytosis. The size of clathrin-coated pits varies between different cell types within the same species [33]. The size of clathrin-coated vesicles depends on the size of its cargo, having an upper limit of 200 nm external diameter in the case of virus uptake [34].

In a similar way, 50 and 120 nm folate-decorated poly(ethylene glycol)-polycaprolactone nanoparticles were found to be internalized via both clathrin- and caveolae-mediated endocytosis in ARPE-19 cells. However, 250 nm nanoparticles, were only internalized via caveolae-mediated pathway [35], despite some articles have described an upper limit size of ~150 nm for passage through caveolae vesicles [36].

In a more recent article, glycopeptide engineered PLGA nanoparticles, closely related to the PHBV nanoparticles studied here, were investigated to determine their endocytic mechanism. As demonstrated by confocal microscopy analysis, these nanoparticles, 170 nm in diameter, strongly colocalized with clathrin related-but not with caveolin related-routes [37].

**Fig. 1:**
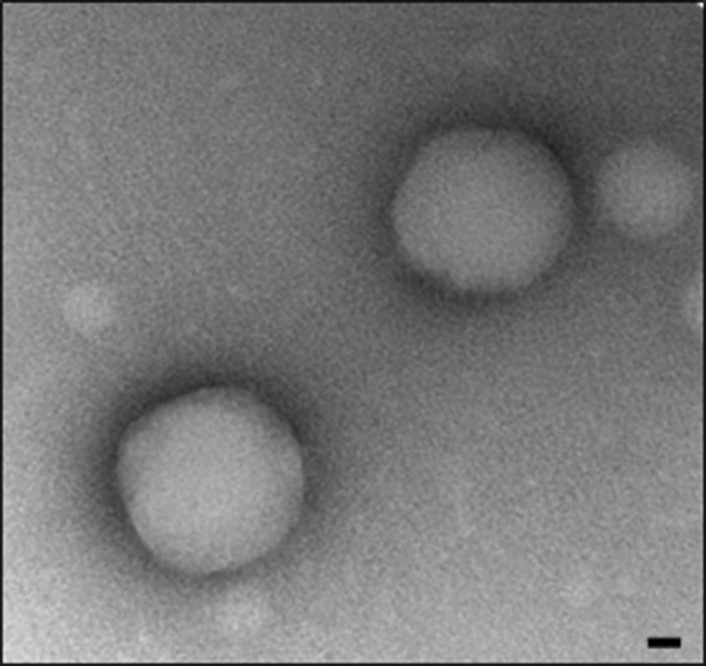
PHBV nanoparticle micrograph. Representative image of PHBV nanoparticles (naked) by transmission electron microscopy (TEM). Objective ×49.000 magnification. *Bar size* 35 nm

Figure 1 shows a representative micrograph of control preparation, which was obtained through transmission electron microscopy (TEM). In this figure, the spherical morphology and uniform nanoparticle size is evidenced. It has been reported that nanoparticle morphology may influence its internalization into the cells. In previous reports, spherical-shaped gold NPs displayed higher and faster rate of endocytosis than disk or rod-shaped nanoparticles [38]. In contrast, other researchers have suggested a preferential internalization of polyethylene glycol nanoparticles having a rod or cylindrical shape [39]. All these records indicate that spherical morphology of PHBV nanoparticles may not be decisive in entering the cells, but the setting of other features—such as the chemical nature, size, and charge—are also implied in how they are taken up.

Surface charge of nanoparticles is another important parameter to characterize because the surface of the nanoparticle interacts directly with the biological environment. Due to the chemical nature conferred by the phospholipids, the plasma membrane has a negative charge. For this reason, positively-charged drug delivery systems exhibit better adhesion and a higher internalization rate. An example of this phenomenon has been demonstrated by Harush-Frenkel et al., who revealed in HeLa cells, that polymeric nanoparticles of PLA-PEG coated with cationic lipid stearylamine (~ +35 mV) evidence greater incorporation than the same NPs but negatively charged (~ −35 mV) [40]. However, in the case of possible use in vivo of these nanoplatforms, the interaction with plasma proteins is reduced if the NPs are negatively charged, decreasing the probabilities of activation of immune response by monocytes and macrophages in the bloodstream [41].

### Uptake measurement of PHBV nanoparticles

To characterize how PHBV nanoparticles are taken up by the cells, we quantified their incorporation through flow cytometry. This measurement was performed under different conditions, including the use of different formulations of NPs, variation of the incubation time, type of cells, and modifying the energy conditions of the cell culture. The latter condition was evaluated because endocytosis is an active cellular process, which requires energy (ATP).

**Fig. 2:**
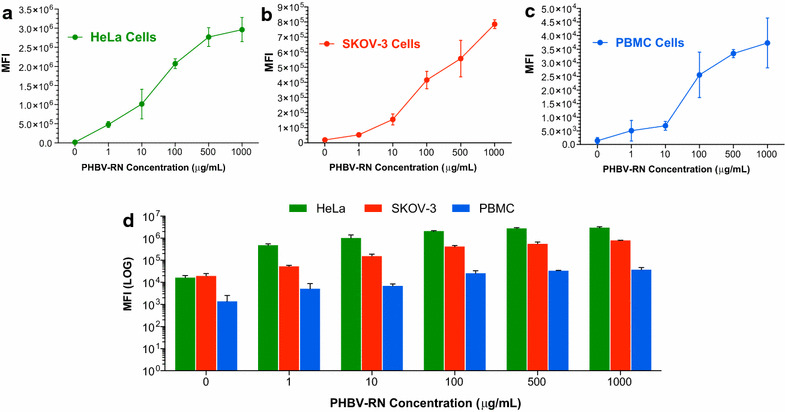
Quantification of the cellular uptake of PHBV nanoparticles: time dependency. To evaluate time dependency in the cellular uptake of NPs, HeLa (**a**), SKOV-3 (**b**) and PBMC (**c**) cells were incubated with a 100 µg/mL PHBV-RN at several times. **d** Mean fluorescence intensity analysis of the three different cells. Mean fluorescence intensity was determined by flow cytometry (n = 3)

The time required for the incorporation of NPs in a cell system is critical to evaluate its use for in vivo applications. While NPs interacts with the plasma membrane, cell will package this “foreign agent” with different surface molecules, allowing their uptake [42]. Generally, by increasing the time which cells are exposed to NPs, their uptake increases. However, this process will be finally determined by the balance between entry and excretion of NPs. Time dependence in NPs incorporation was evaluated in PMBC, HeLa and SKOV-3. As shown in Fig. 2, HeLa (a) and SKOV-3 (b) show time-dependence, as evidenced by a high mean fluorescence intensity (MFI) given by the encapsulated fluorophore, showing a similar behavior obtained by Liu et al. [25]. Nevertheless, the same phenomenon was not observed in PBMC. After 5 min of incubation, a high fluorescence intensity was observed, which did not vary under longer incubation, reaching stable values. Most cellular models used so far to investigate nanoparticle cellular uptake do not take into account cellular heterogeneity in the blood stream, for instance, the presence of macrophages or neutrophils. Differences in NPs uptake and/or NPs phagocytosis can be found even between macrophages mouse strains dependent on their preference for either Th1- or Th2-responses [43]. The mentioned phenomena may explain why in our experiments performed with PBMC, we observed a faster NPs internalization compared to the other cell lines (Fig. 2c, d).

**Fig. 3:**
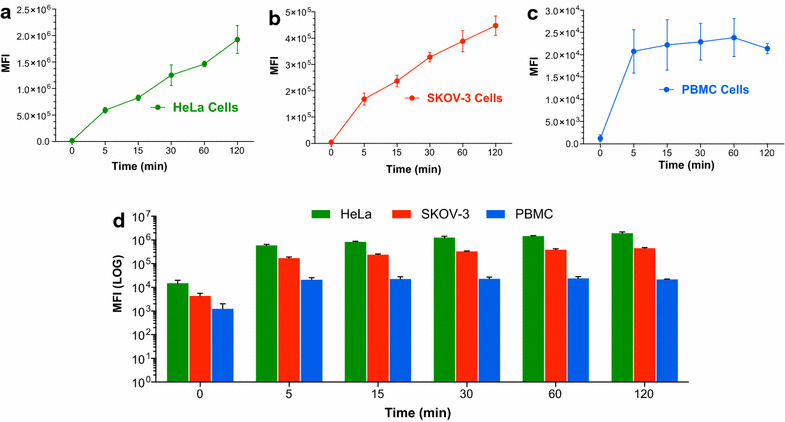
Quantification of the cellular uptake of PHBV nanoparticles. Concentration dependency. To evaluate concentration dependency in the cellular uptake of NPs, HeLa (**a**), SKOV-3 (**b**) and PBMC (**c**) were incubated with a PHBV-RN solution at different concentrations (1, 10, 100, 500 and 1000 µg/mL) for 2 h. **d** Mean fluorescence intensity analysis of the three different cells. Mean fluorescence intensity was determined through flow cytometry (n = 3)

NPs concentration is another important feature to be analyzed because it can impact uptake efficiency and cytotoxicity induction. Thus, we evaluated concentration dependence in the uptake of PHBV nanoparticles. HeLa, SKOV-3 and PBMC cells were incubated for 2 h at 37 °C, 5% CO_2_ with a solution of PHBV-RN at different concentrations: 1, 10, 100, 500 and 1000 µg/mL. Figure 3a–d shows that in all the three cell types, concentration-dependence is observed, with an apparent saturation at 500 µg/mL. Experiments performed in PBMC indicate that cells need a specific concentration to internalize NPs, although they can internalize less content compared to the other cell lines.

PBMC showed faster cellular uptake in time (comparing with the other cells at 5 min). This phenomenon was not observed in the other cell lines probably because PBMC contains macrophages and neutrophils which endocytosis mechanism is most via phagocytosis [43]. Nevertheless, uptake after 5 min seems to be inefficient probably because PBMC are smaller cells compared to the other cell lines (HeLa and SKOV-3 cells are relative equal in size). This idea is supported by Fig. 2d, which shows that PBMC uptake with different concentrations reach a plateau, absorbing less NPS than the other cell lines. On the other hand, cell lines (which are derived from a tumor) display a high metabolic rate, process that can respond to a higher cellular uptake without reaching a plateau. This effect was more evident in HeLa cells than SKOV-3 cells.

We have estimated an approximate number of NPs at a given NP mass concentration depicted in Fig. 3, following the previous data from Vilos et al. [32]. In this way, the fluctuation in the number of NPs at 500 µg/mL compared to 1000 µg/mL represents only a twofold increase in picomoles/mL. It must be noted that an increase in the concentration of the solution might not be “saturating” the cells, because the NPs could enter progressively. Probably, at a high solution concentration such as 1000 µg/mL, NPs can enter in similar extent than at 500 µg/mL to the cells, as Fig. 3 shows, but probably afterwards, every NP can eventually enter the cells.

**Fig. 4:**
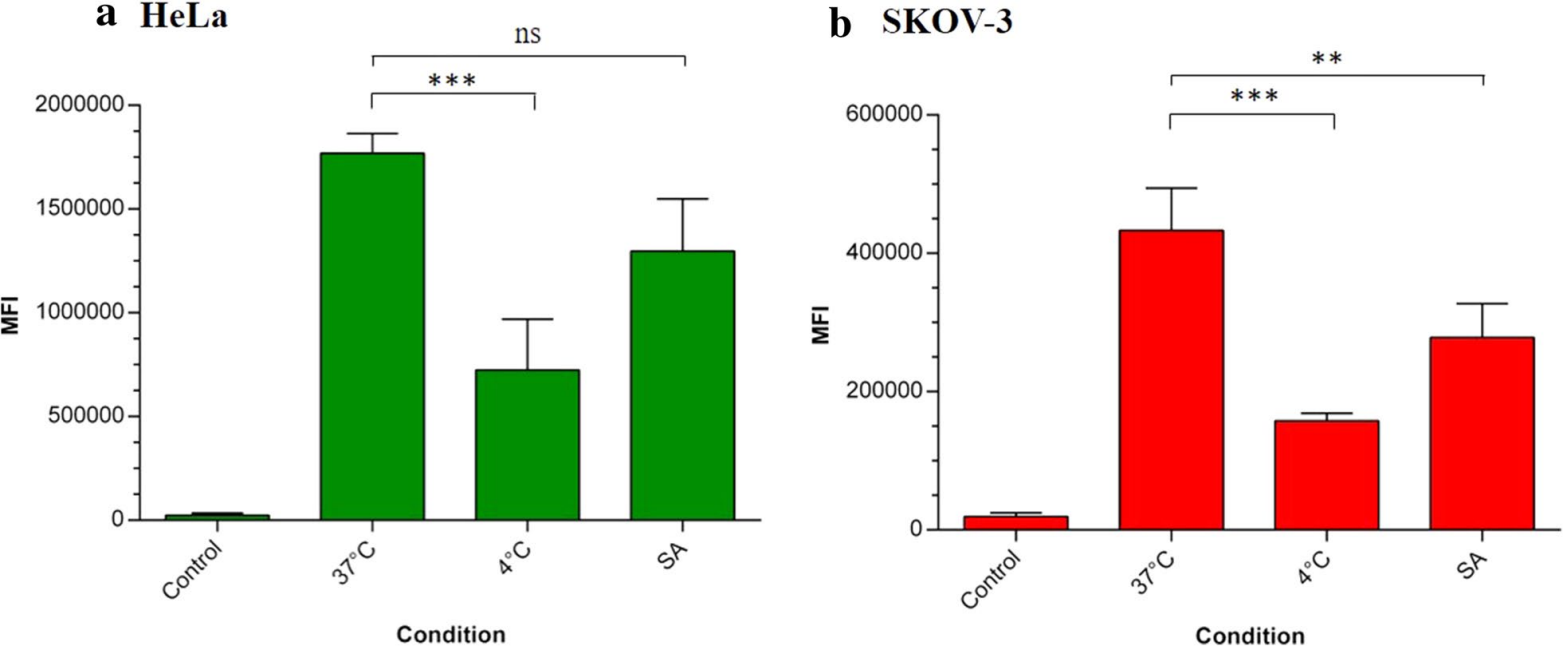
Quantification of the cellular uptake of PHBV nanoparticles: energy dependency. To evaluate energy dependency in the cellular uptake of NPs, HeLa (**a**) and SKOV-3 cells (**b**) were incubated with 100 µg/mL PHBV-RN under different conditions (37, 4 °C, sodium azide). Mean fluorescence intensity was determined through flow cytometry (n = 3). One-way ANOVA and Bonferroni statistical test were used (**p < 0.01, ***p < 0.001, ****p < 0.0001)

Internalization and processing of nanoparticles into the cells is an active process, which requires energy to be performed [44]. Dependence of energy (i.e. ATP) was assessed in HeLa and SKOV-3 cells. Both cell lines were incubated at 4 °C, after that the uptake of nanoparticles was quantified. As seen in Fig. 4, when both cell lines were incubated at 4 °C a decrease of NPs internalization was observed, suggesting endocytosis inhibition. Other groups also have reported this behavior. Qaddoumi et al. [20] reported a decrease of about 90% in the uptake of PLGA nanoparticles in rabbit conjunctival epithelial cells (RCEC), while Liu et al. [25] also observed a decrease in uptake of polymeric nanoparticles in human cell lines Hep-G2, HuH-7, and PLC. By the other hand, treatment with sodium azide (SA) solution, which blocks electron transport chain, showed a decrease of the entry of NPs, however, this decay is less significant than the observed at 4 °C. One possible explanation for this phenomenon is that SA inhibits synthesis of ATP, then cells may use exogenous ATP for operation, allowing entry of PHBV nanoparticles, as been suggested by Gratton et al. [39]. Other researchers have evaluated inhibition of endocytosis adding sodium azide, obtaining similar results [45]. All these results suggest that uptake of PHBV nanoparticles is a time, concentration, and energy-dependent process.

To study the effect of NPs on cell viability, we analyzed cell viability with annexin V by flow cytometry and caspase-3 by immunofluorescence. Results show no significant levels of cytotoxicity induced by the NPs in HeLa and SKOV-3, which was demonstrated using different assays and different concentrations and incubation times (Additional file 3: Figure S3).

### Endocytosis mechanism of PHBV nanoparticles

**Fig. 5:**
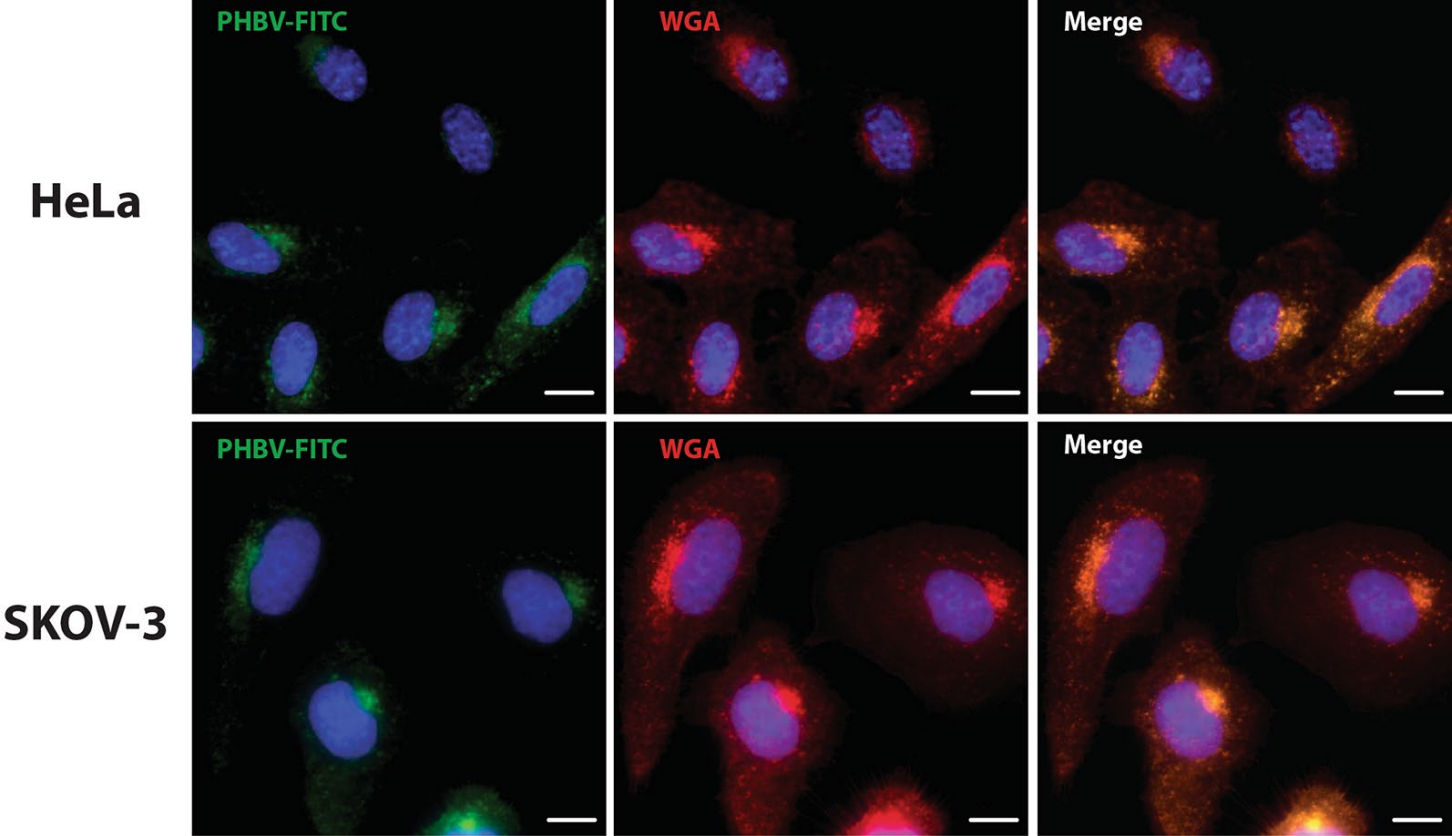
Characterization of the endocytosis mechanism using WGA marker. HeLa and SKOV-3 cells were incubated with PHBV-FITC for 5 min at 37 °C, in the presence of Alexa Fluor® 555-conjugated WGA. Later, cells were fixed and observed by fluorescence microscopy. Hoechst 33342 was used as a nuclear stain. Objective: ×60 magnification. *Bar size* 10 µm

To determine endocytosis mechanism of PHBV nanoparticles, we used fluorescent marker WGA, which selectively recognizes plasma membrane structures. HeLa and SKOV-3 cell lines were incubated for 5 min with a PHBV-FITC solution and subsequently fixed for analysis. In Fig. 5 is observed colocalization of nanoparticles with WGA marker (arrowheads), demonstrating that uptake is performed by a plasma membrane coated compartment in both cancer cell lines (HeLa 99.98% and SKOV-3 99.95% of colocalization).

**Fig. 6:**
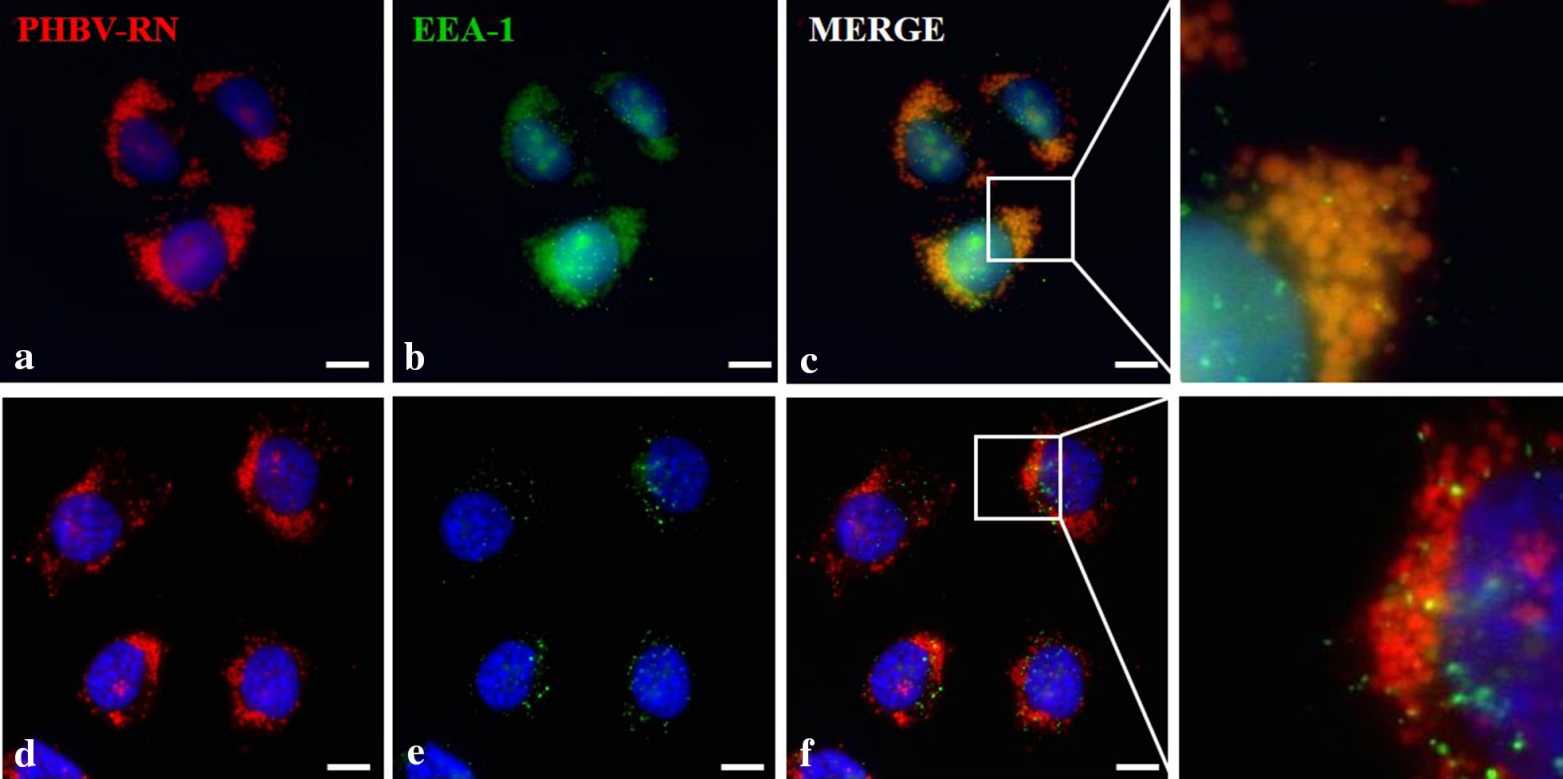
Characterization of the endocytosis mechanism using EEA-1 marker. HeLa (**a**–**c**) and SKOV-3 (**d**–**f**) cells were incubated with PHBV-RN nanoparticles for 15 min at 37 °C; then cells were fixed and permeabilized. Later, an immunofluorescence was performed using anti-EEA1 (1:100) and anti-mouse Alexa Fluor® 488 (1:500) as primary and secondary antibody respectively. Finally, cells were observed through fluorescence microscopy. Hoechst 33342 was used as nuclear stain. Objective: ×60 magnification. *Bar size* 10 µm

**Fig. 7:**
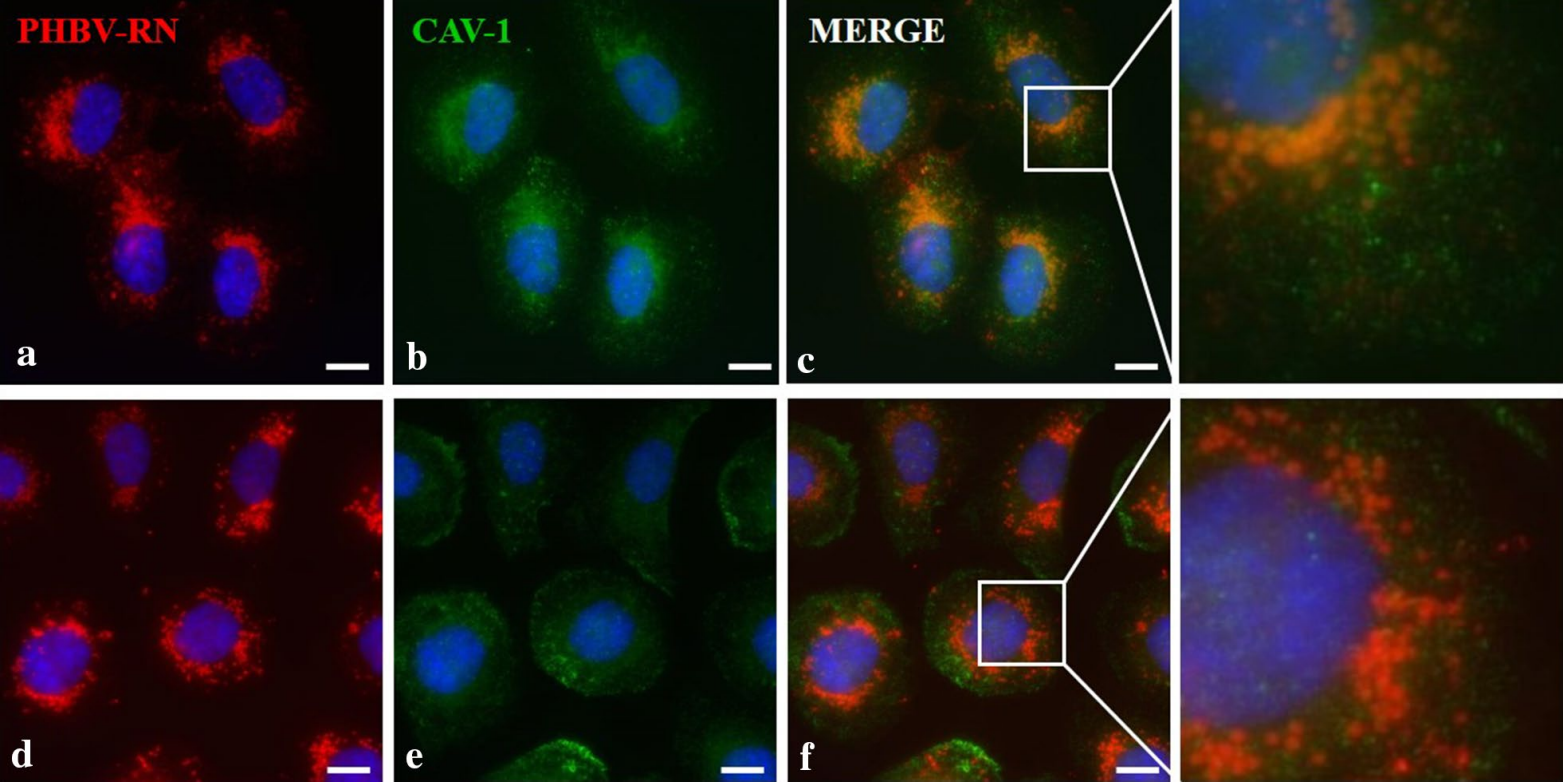
Characterization of the endocytosis mechanism using CAV-1 marker. HeLa (**a**–**c**) and SKOV-3 (**d**–**f**) cells were incubated with PHBV-RN nanoparticles for 15 min at 37 °C, then cells were fixed and permeabilized. Later, an immunofluorescence was performed using anti-CAV1 (1:100) and anti-mouse Alexa Fluor® 488 (1:500) as primary and secondary antibody respectively. Finally, cells were observed through fluorescence microscopy. Hoechst 33342 was used as nuclear stain. Objective: ×60 magnification. *Bar size* 10 µm

As a next step, we assessed whether entry of PHBV NPs is done through classical endocytosis ways. For this purpose, EEA-1 (early endosomes marker) (Fig. 6) and CAV-1 (caveolae-mediated endocytosis marker) (Fig. 7) were used. Both cell lines were exposed for 5 and 15 min to PHBV-RN solution and then fixed. As shown in Fig. 6, HeLa cell line shows clear colocalization of NPs with early endosomes showing 99.42% of colocalization at 5 min and 99.39% of colocalization at 15 min, while SKOV-3 cells colocalization is strongly lesser showing 21.17% of colocalization at 5 min and 23.08% of colocalization at 15 min. Furthermore, Fig. 7 shows in HeLa cells some colocalization of PHBV nanoparticles with endosomes coated with caveolae (41.46% of colocalization at 5 min and 30.62% of colocalization at 15 min). While in SKOV-3 cells, colocalization with CAV-1 was not observed, evidencing that PHBV NPs do not enter the cells through caveolae-coated vesicles, although SKOV-3 have been described as one of the few ovarian carcinoma cells expressing caveolin-1 protein [46]. Experiments conducted by Ekkapongpisi et al., showed that negative-coated silica nanoparticles were uptaken by a caveolin-independent mechanism by SKOV-3 cells, which was similar to neutral PHBV nanoparticles described with mesoporous silica and polystyrene nanoparticles [47].

In contrast to clathrin-mediated, classic route, caveolar uptake has been suggested to avoid lysosomes. Caveolae vesicles fuse with caveosomes, delivering their content to other cellular compartments. By avoiding acidic routes, a caveolar pathway may be advantageous for drug delivery. In this manner, ligands can be developed to target caveolar domains and reaching a more efficient delivery [48].

**Fig. 8:**
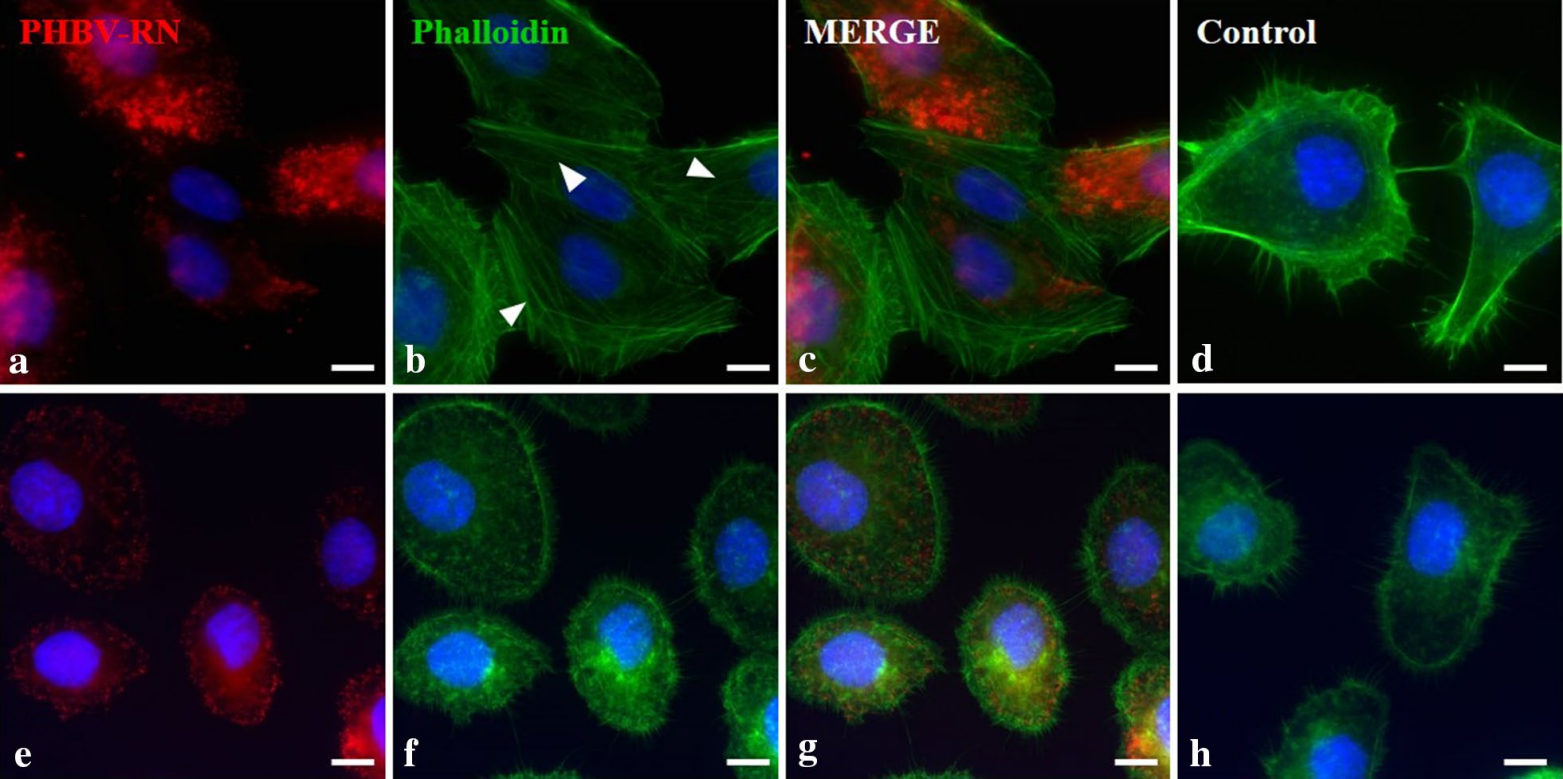
Evaluation of actin filaments rearrangement in the presence of PHBV nanoparticles. HeLa (**a**–**d**) and SKOV-3 (**e**–**h**) cells were incubated with PHBV-RN for 15 and 30 min at 37 °C; then cells were permeabilized and incubated with Phalloidin Alexa Fluor® 488 conjugated. Hoechst 33242 was used as nuclear stain. Objective: ×60 magnification. *Bar size* 10 µm

Macropinocytosis mechanism was also indirectly evaluated. This pathway is characterized by the rearrangement of actin filaments in the cytoplasm when a particle is incorporated. As a marker of actin monomers, Phalloidin, which is a mycotoxin that binds irreversibly to actin monomers, was conjugated to Alexa Fluor® 488 fluorophore. HeLa and SKOV-3 cell lines were incubated for 15 and 30 min with a PHBV-RN solution. Figure 8 shows that HeLa exhibits stress fibers in the presence of PHBV nanoparticles (arrowheads). These fibers are characteristic of rearrangement of actin filaments in cellular processes such as macropinocytosis. It is important to note that in SKOV-3, these types of fibers are not seen, showing the same form as control treatment.

In summary, NPs showed different cell entry ways depending on the cell type; specifically, in SKOV-3, NPs appear to enter through an independent way from the traditional routes, unlike the behavior observed in HeLa cells (summary of the colocalization analysis is given in Table 2).

**Table 2:** Extent of colocalization of PHBV nanoparticles with endocytosis markers in HeLa and SKOV-3 cells

	CAV-1		EEA-1		WGA
	5 min	15 min	5 min	15 min	5 min
HeLa (%)	41.46	30.62	99.42	99.39	99.98
SKOV-3 (%)	0	0	21.17	23.08	99.95

**Fig. 9:**
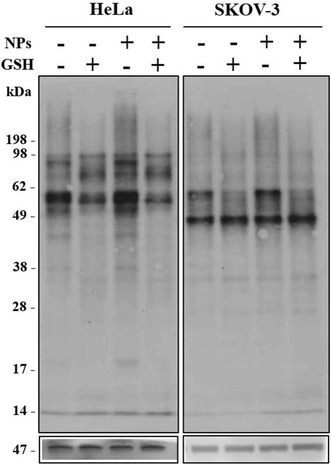
Analysis of proteins involved in cellular uptake of PHBV nanoparticles. HeLa and SKOV-3 cells were subjected to reducible biotin assay to determine possible membrane proteins involved in nanoparticles uptake. 40 µg of proteins from cell lysate were resolved through SDS-PAGE, then transferred to PDVF membrane and finally, incubated with streptavidin-HRP (1:1000). *β*-actin (47 kDa) was used as loading control

To determine the participation of the proteins from the cell membrane in the uptake of NPs, a reducible biotin assay was performed. By using a reducing agent such as glutathione, this method allows discriminating surface proteins that enter into the cell under a certain condition. As shown in Fig. 9, apparently there were no specific proteins involved in the incorporation of nanoparticles, because no differences were observed in the protein pattern obtained with NPs compared to the control condition without treatment. However, it is important to remark that the protein membrane pattern evaluated by western blot is completely different between both cell lines, suggesting that endocytosis machinery and plasma membrane proteins together with its dynamics may be different among them. This observation could explain our immunofluorescence results that showed that actin filaments, which are the major cytoskeletal proteins, are not acting in the NPs entry to SKOV-3 cells, differently to Hela cells.

**Fig. 10:**
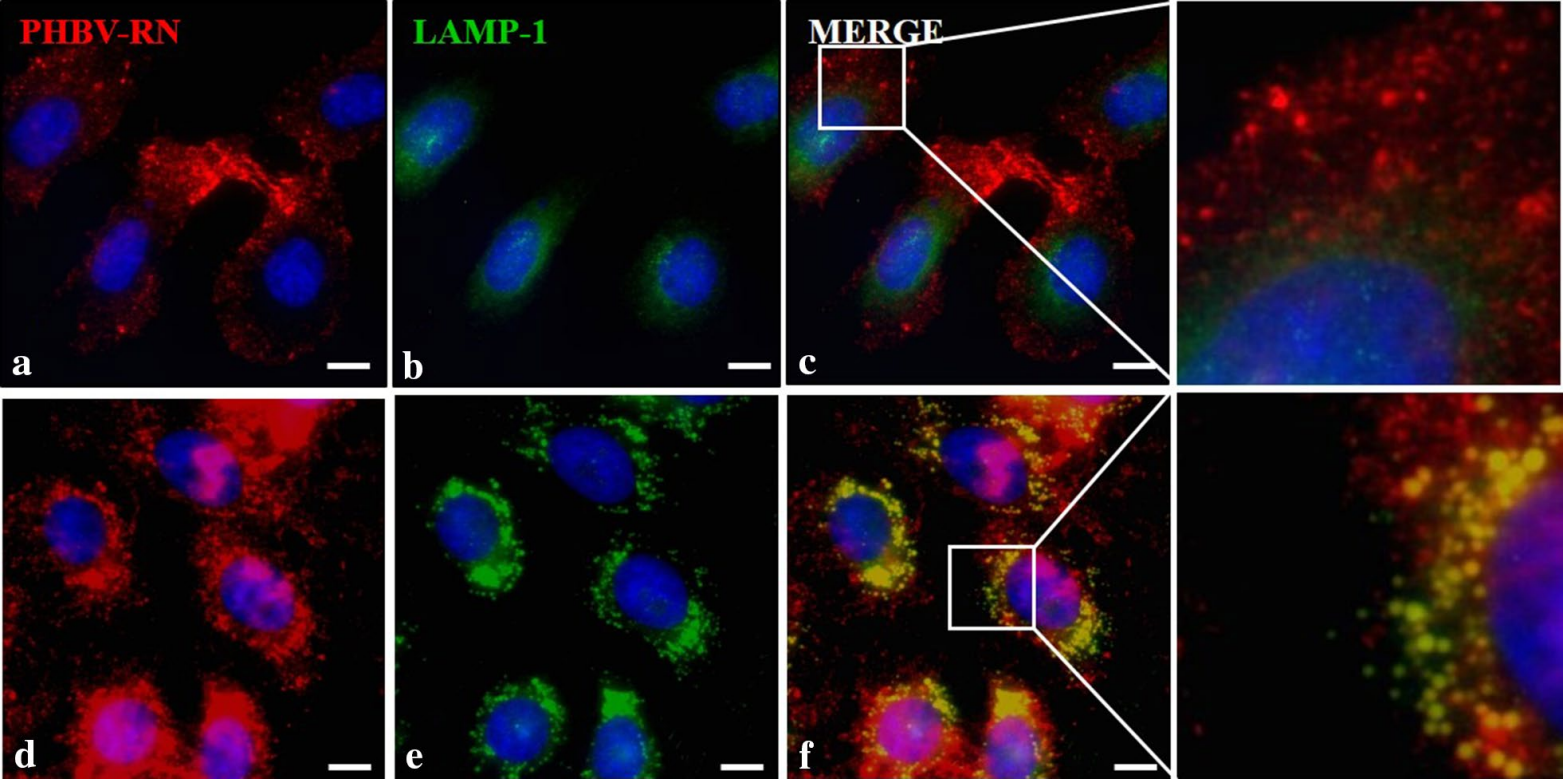
Determination of final nanoparticle fate in HeLa cells. HeLa cells were incubated with PHBV-RN nanoparticles for 15 min (**a**–**c**) and 1 h (**d**–**f**) at 37 °C; then cells were fixed and permeabilized. Later, an immunofluorescence was performed using anti-LAMP1 (1:100) and anti-mouse Alexa Fluor® 488 (1:500) as primary and secondary antibody respectively. Finally, cells were observed through fluorescence microscopy. Hoechst 33342 was used as nuclear stain. Objective: ×60 magnification. *Bar size* 10 µm

**Fig. 11:**
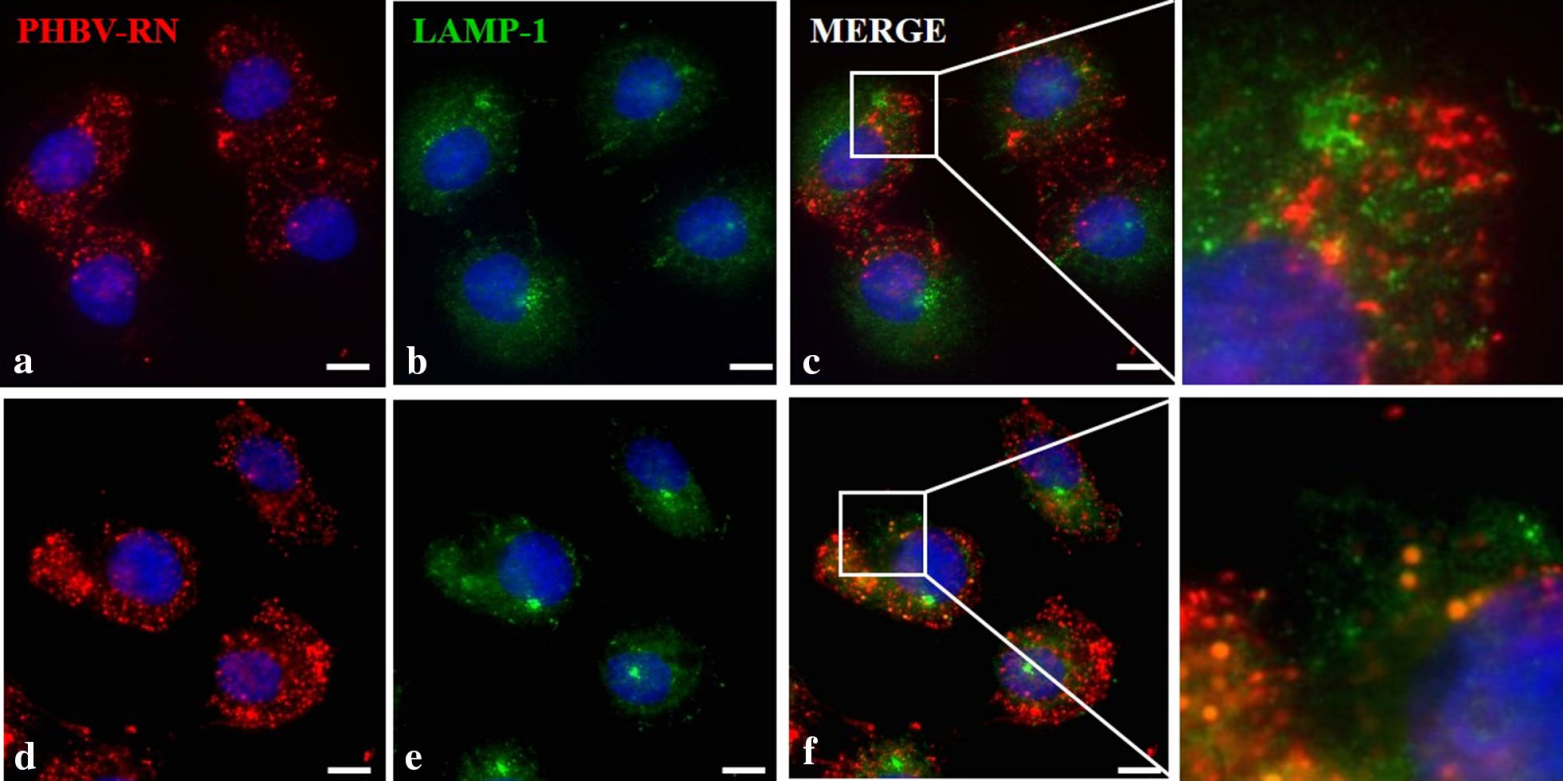
Determination of final nanoparticle fate in SKOV-3 cells. SKOV-3 cells were incubated with PHBV-RN nanoparticles for 15 min (**a**–**c**) and 1 h (**d**–**f**) at 37 °C, then cells were fixed and permeabilized. Later, an immunofluorescence was performed using anti-LAMP1 (1:100) and anti-mouse Alexa Fluor® 488 (1:500) as primary and secondary antibody respectively. Finally, cells were observed through fluorescence microscopy. Hoechst 33342 was used as nuclear stain. Objective: ×60 magnification. *Bar size* 10 µm

Furthermore, to determine the final fate of the NPs in both cell lines, colocalization with LAMP-1 (Lysosome-Associated Membrane Protein 1) antibody was performed by immunofluorescence (Figs. 10, 11), showing that in both cases NPs do colocalize with lysosomes but at different times: in HeLa cells, transit towards lysosomes was faster than in SKOV-3, probably due to that in Hela, the classical entry pathway is preferred by the NPs, allowing more expedite transit to the lysosomes, unlike SKOV-3. Moreover, in experiments performed with HL-60, a human promyelocytic leukemia cell line, after 4 h of incubation with NPs containing the fluorophore FITC, its content was observed in the cytoplasm meaning that somehow NP content may be released by the lysosomes where NPs may be degraded (data not shown).

**Fig. 12:**
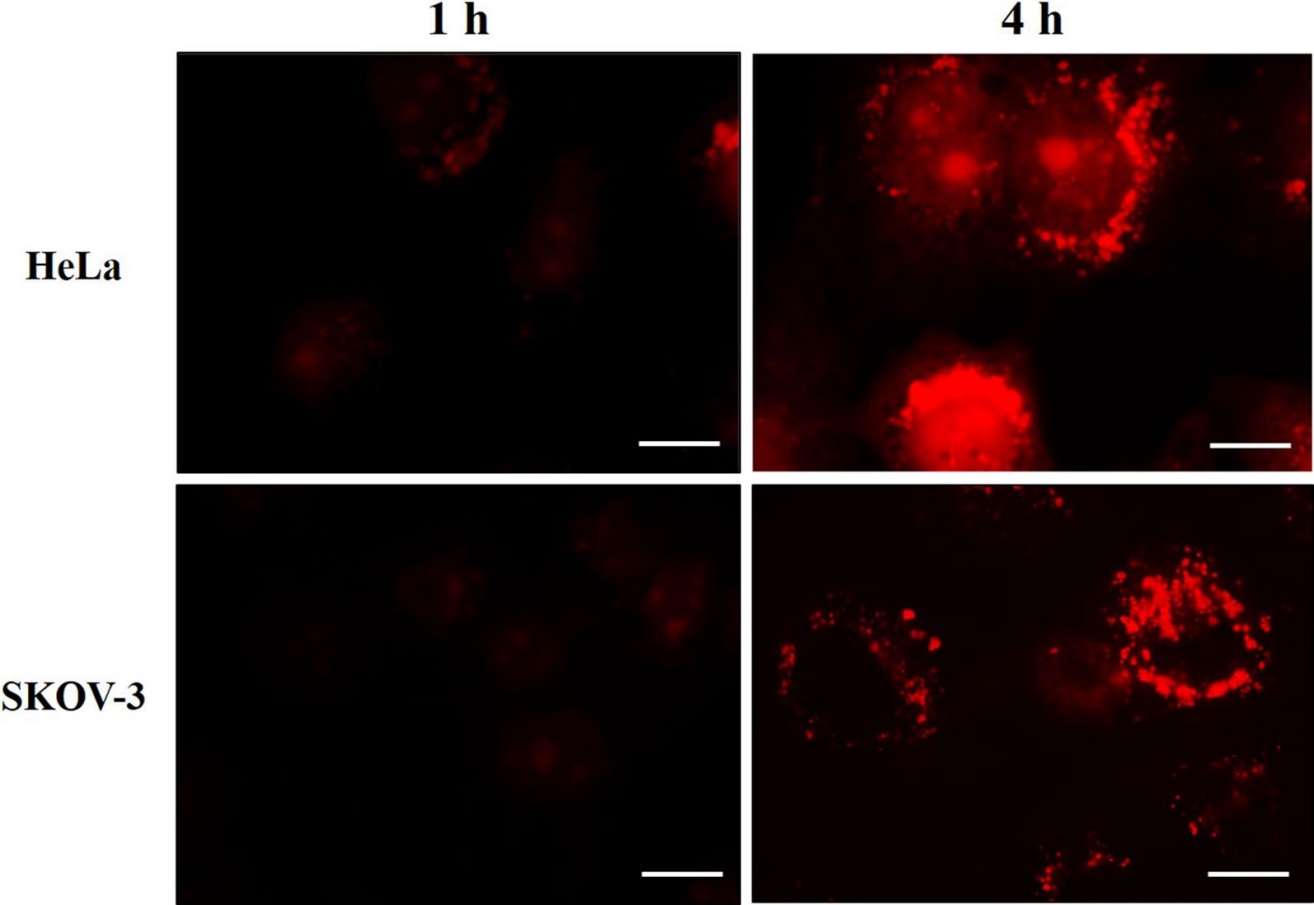
Evaluation of nanoparticle degradation. HeLa and SKOV-3 cells were incubated with PHBV-Lysotracker® DND-99/nanoparticles for 4 h at 37 °C, then cells were fixed and observed through fluorescence microscopy. Objective: ×60 magnification. *Bar size* 10 µm

To evaluate NPs final fate through a different approach, we performed an experiment with NPs encapsulating LysoTracker® probe, which consist on a fluorophore linked to a weak base that is only partially protonated at neutral pH [49]. This probe is highly selective for acidic organelles. If the NPs release their content in the lysosome, it may be detected by fluorescence microscopy. In this way, fluorescence is not detected when LysoTracker® is trafficking through the endosomes. Our results in Fig. 12 shows that after 4 h, fluorescence can be detected, demonstrating that NPs are degraded in the lysosome, releasing their content. In vitro degradation of PHBV has been shown very slow [50]. At extreme conditions, such as at acid/basic pH, degradation seems to undergo by a decrease in the molecular weight and then, by a weight loss, when the mechanical strength is lost, and the remaining PHBV breaks down into small fragments [51].

**Fig. 13:**
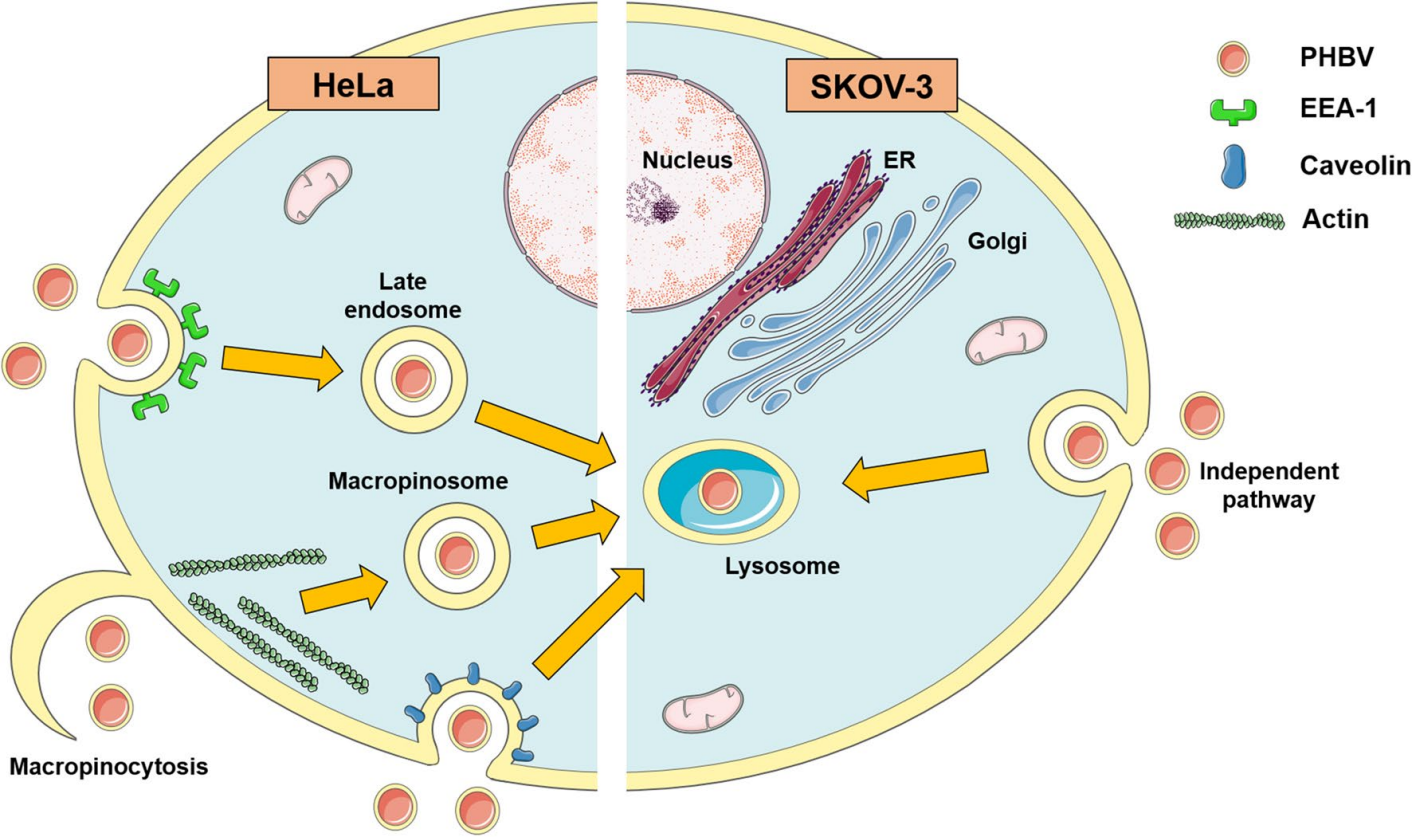
PHBV nanoparticle intracellular trafficking pathway in HeLa and SKOV-3 cells scheme. Our results showed different endocytosis pathway depending on the cell context but the same final destination

When cells were incubated with PHBV NPs, after trafficking through the endo-lysosome pathway, which finally ends at lysosomes, these organelles enter into a special stage where they fused and get polarized, resembling what normally happens during autophagy and immunological synapsis [52, 53]. To investigate whether NPs/cell incubation may trigger autophagy, we analyze LC3 expression by western blot in the two different cell lines, which showed no LC3 expression (data not shown) suggesting that lysosomal fusion not be triggering by an autophagy event [54]. These findings lead to propose the recruitment of a possible MTOC (Microtubule Organizing Center) towards a particular area, allowing the local regulation of potential exocytosis and endocytosis processes by concentrating at a specific place the required molecular machinery. A scheme of PHBV NPs entry pathway for HeLa and SKOV-3 cell lines suggested in this article is depicted in Fig. 13.

## Conclusion

We investigated the cellular uptake mechanism of PHBV for intracellular delivery into HeLa and SKOV3 cells. Our experimental results showed that this process is time, concentration and energy-dependent, and the internalization mechanism depends on the cell line. Results described above, give us a general understanding of the cellular processes required for nanoparticle internalization, which contributes to understanding further targeting properties of PHBV enhancing their targeting efficiency.

### Additional files

**Additional file 1: Figure S1.** Physicochemical characterization of nanoparticles. A) Size, B) Z Potential and C) Polydispersion Index.

**Additional file 2: Figure S2.** PHBV nanoparticles stability. Synthesized nanoparticles were stored at 4 °C for four week period and then analyzed by DLS (Size and zeta potential). Results are expressed as the mean ± standard deviation of triplicate determinations from three independent experiments. One-way ANOVA with Bonferroni test as statistical analysis was performed. ns = not significant, * P <0.05, ** P <0.01, *** P <0.001.

**Additional file 3: Figure S3.** Cytotoxicity of PHBV nanoparticles at 1, 10, 100 and 1.000 μg/mL against HeLa cells using the MTT assay. Cells were incubated with the respective concentrations and left untreated to measure cell viability by MTT assay.
