# Supplementary figures and images for: Intracellular trafficking and cellular uptake mechanism of PHBV nanoparticles for targeted delivery in epithelial cell lines
Source: J Nanobiotechnology. 2017 Jan 3;15:1. doi: 10.1186/s12951-016-0241-6 (PMC5210312; doi:10.1186/s12951-016-0241-6)

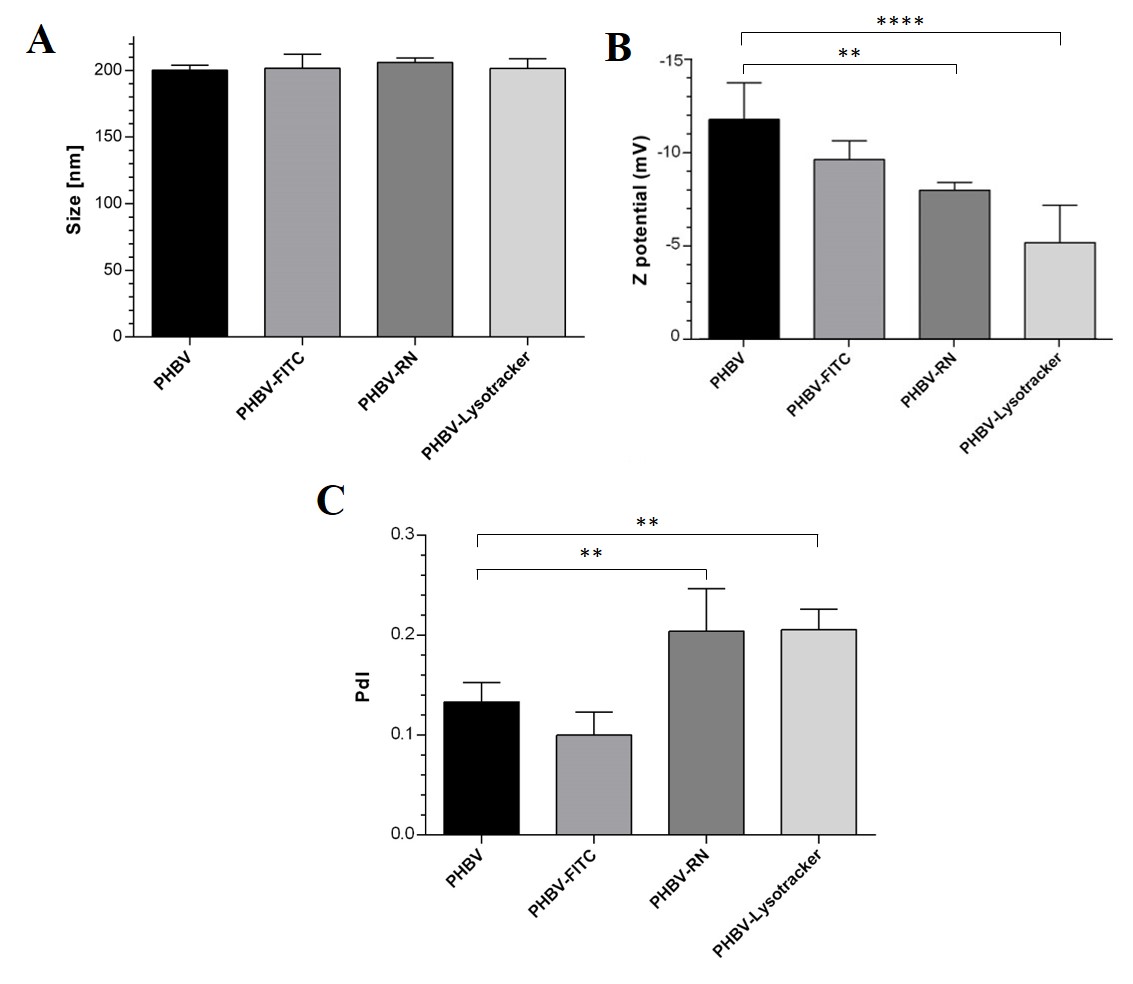

Supplement: Supplementary file 1 — Additional file 1: Figure S1. Physicochemical characterization of nanoparticles. A) Size, B) Z Potential and C) Polydispersion Index. [file 12951_2016_241_MOESM1_ESM.jpg]

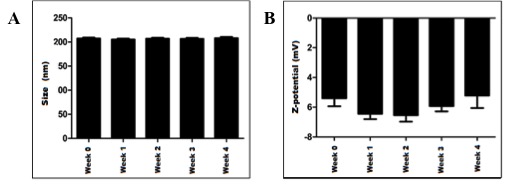

Supplement: Supplementary file 2 — Additional file 2: Figure S2. PHBV nanoparticles stability. Synthesized nanoparticles were stored at 4 °C for four week period and then analyzed by DLS (Size and zeta potential). Results are expressed as the mean ± standard deviation of triplicate determinations from three independent experiments. One-way ANOVA with Bonferroni test as statistical analysis was performed. ns = not significant, * P <0.05, ** P <0.01, *** P <0.001. [file 12951_2016_241_MOESM2_ESM.jpg]

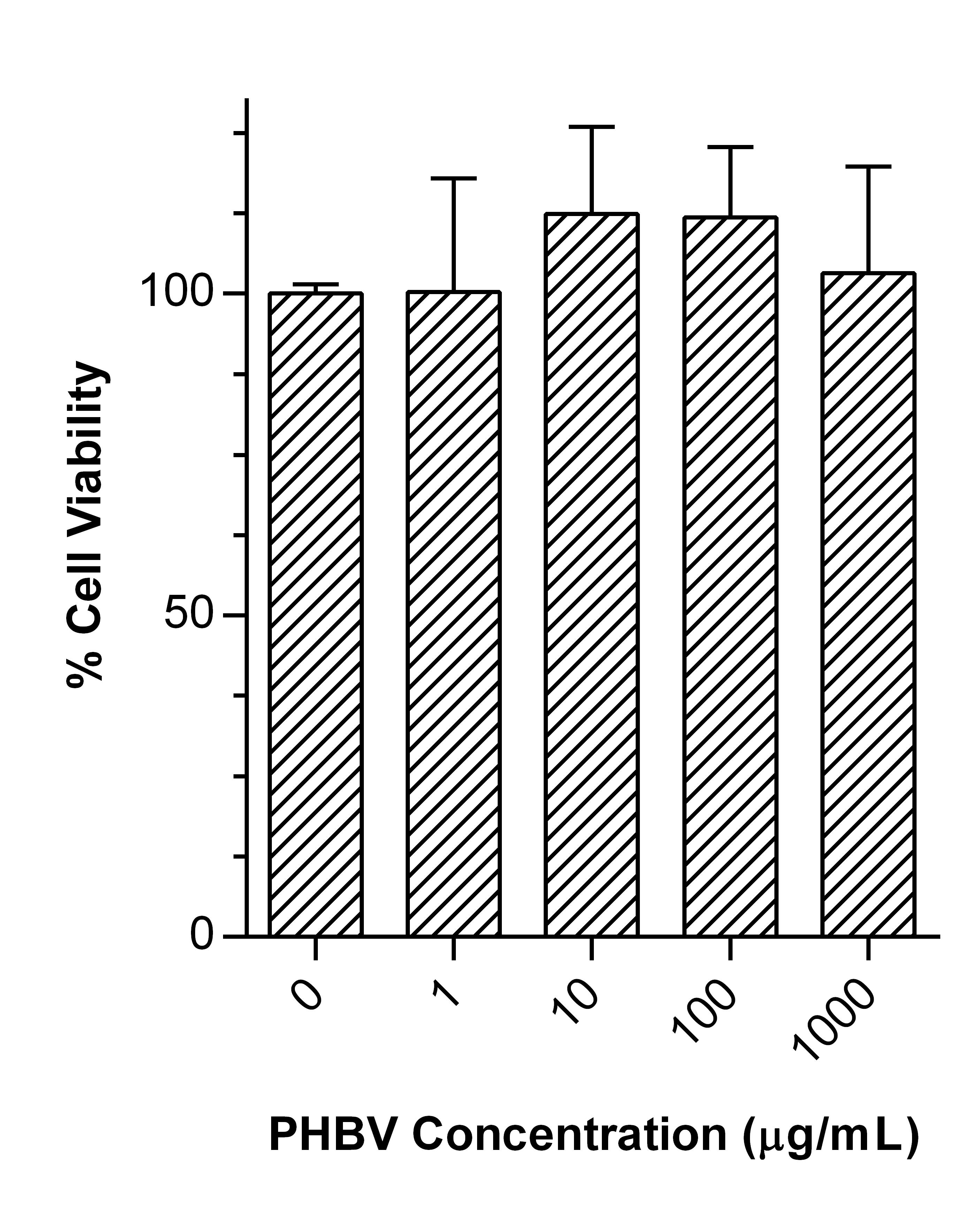

Supplement: Supplementary file 3 — Additional file 3: Figure S3. Cytotoxicity of PHBV nanoparticles at 1, 10, 100 and 1.000 μg/mL against HeLa cells using the MTT assay. Cells were incubated with the respective concentrations and left untreated to measure cell viability by MTT assay. [file 12951_2016_241_MOESM3_ESM.jpg]
